# Who is responsible for providing care? Investigating the role of care tasks and past experiences in a cross-sectional survey in the Netherlands

**DOI:** 10.1186/s12913-017-2435-5

**Published:** 2017-07-11

**Authors:** R. J. Hoefman, T. M. Meulenkamp, J. D. De Jong

**Affiliations:** 1 Institute of Health Policy and Management, Erasmus University Rotterdam, PO Box 1738, 3000 DR Rotterdam, Rotterdam, The Netherlands; 2 Netherlands institute for health services research, PO Box 1568, 3500 BN Utrecht, Utrecht, The Netherlands; 3 Department of Health Services Research, Maastricht University, PO Box 616, 6200 MD Maastricht, Maastricht, The Netherlands

**Keywords:** Informal care, Responsibility for care, Views on care, Care tasks, Previous experiences with care, Family care

## Abstract

**Background:**

Many countries face substitution from formal to informal care. It is essential that a sufficient number of caregivers, such as family, friends or neighbors, are willing and able to lend care to address the needs of ill or elderly persons. We investigated whether the general public, who might become caregivers in the future, and current informal caregivers align with the shift to more informal caregiving.

**Methods:**

We studied the views on the responsibility for care of the general public versus the government, and whether these views differed among groups with diverse past experiences with care in terms of own health problems or previous caregiving activities. Data (*n* = 1097) was collected among the Dutch Health Care Consumer Panel with a survey in October 2015. Multivariate analyses of the views on responsibility for care in general and for different types of care were performed using (i) health, (ii) informal care, and (iii) general background characteristics, among a sample of the general public and among a subgroup of current caregivers.

**Results:**

The majority (67%) of the respondents would be willing to provide informal care in the future, when necessary. Respondents were more willing to provide support tasks than personal or nursing care activities. Among current caregivers, views on responsibility for care were associated with their past experience. Experiencing less burden of caregiving was associated with perceiving the general public as more responsible for personal or nursing care.

**Conclusions:**

The results of this study show that substitution from formal to informal care is more in line with public views when support activities are concerned than personal or nursing care. In addition, burdened caregivers also consider the government more responsible for personal or nursing care. When handing over care tasks to the public domain a critical view is needed on which care tasks are most appropriate for this.

**Electronic supplementary material:**

The online version of this article (doi:10.1186/s12913-017-2435-5) contains supplementary material, which is available to authorized users.

## Background

To fulfill the needs of chronically ill or elderly persons it is essential that a sufficient number of family, friends or neighbors are willing and able to lend care. From the literature, it is well-known that informal caregiving can be a fulfilling activity, but can nevertheless be burdensome for caregivers, especially when they need to combine their care activities with paid work or family life. Caring can both be emotionally and physically demanding and result in health problems [1–11]. As caregiving can be straining, it can be questioned whether the general public is willing to provide informal care in the future anticipating the further increased need for informal care. Western countries are confronted with cutbacks in formal health care spending [12]. To reduce the pressure on the health care budget, governments may substitute some parts of formal health care services for informal care. Next to budget cuts, different demographic developments in Western countries, such as the rapid increase of elderly persons or persons with a chronic disease, and developments in the labour market, such as a scarcity of health care personnel, also increase the need for informal care [13, 14]. In many Western countries, informal caregivers already play an important role in health care with an estimated 5 to 30% of the adult population active as informal caregivers in many Western countries [1, 13, 15–18]. In the Netherlands, about one in three persons of the adult general public lends care to a person needing care from their social network [1]. Around 30% of these caregivers have been providing care for more than three months and for more than eight hours per week. Females and persons aged between 45 to 64 years often are caregivers lending care to parents(−in-law) usually given physical health problems or dementia. Although less prevalent, caregivers also provide care to their partner, child or friends. Caregiving consists of a range of activities, such as emotional support, practical assistance with transport, help with administration and household activities. Caregivers also provide personal or nursing care tasks, but usually these are only provided to close family member, such as a partner or a child [1].

Whether people provide informal care depends on several factors, including the probability of having persons needing care in your network [1]. Moreover, motivations to be socially active are also important, with feelings of obligations, intergenerational affection, filial responsibility and moral duty being especially important for the decision to become an informal caregiver [19–21]. Studies that focus on the availability of informal caregivers are often quite positive on the number of persons available to provide informal care in the future, especially for elderly persons [14, 22, 23]. Predictions of the ‘potential of informal care’ are usually based on the size of the social network of elderly persons or on demographic figures and expected societal changes, for example the number of women in a country and their employment participation rate [22–24]. However, these predictions do not take into account whether people are actually willing to lend care. While studies on the actual provision of informal care are numerous, there is much less research on views on whether the government or the social network should provide care [1, 25, 26]. Studies in Europe and the VS have shown that although there seems to be a transition in views on who should provide care from the government to the social network, many people consider mainly the government to be responsible for care provision [20, 27–29]. These studies usually studied the views of recipients of care [27, 28], current caregivers [26] or concentrated on care to specific social relations. Generally, intergenerational care between (adult) children and their (elderly) parents is the main subject of interest in these studies [27–34]. Others also studied future caregiving by adult siblings of individuals with mental disabilities [35, 36]. While care for close relatives is quite prevalent, informal care is nevertheless not restricted to this type of care. In this paper, we study which views the general public in the Netherlands hold on the responsibility for care for persons with a need for care in their social network. We will focus on views on different types of care tasks, because characteristics of the care situation, such as the type of care tasks that caregivers will perform, also seem to influence care preferences [1, 25]. We will investigate whether people hold different views on responsibility for care. Central in this study is the role of individual characteristics, more specific the role of own experiences of persons on their views on care. It is known from previous research that people evaluate welfare policies differently according to their past experiences. According to the self-interest argument in welfare state research, vulnerable groups that are more likely to need public services in the future, such as low income groups, tend to prefer more comprehensive state based welfare policies [37]. In the context of policies in health care, previous research indicates that preferences for care are shaped by their past experiences with care needs or health care use [27, 38]. When people perceive health problems themselves, they are less likely to participate in caregiving [39] and more often perceive the government responsible for care provision [27, 29]. In this study, we hypothesize that persons who perceive health problems will address more responsibility to the government than to the general public. We also focus on past experiences with informal care. We expect that past experiences of caregivers may also shape views on who should be responsible for care. We hypothesize that caregivers experiencing less burden will address more responsibility for care to the general public instead of the government.

## Methods

### Data

Data was collected among members of the Dutch Health Care Consumer Panel of the Netherlands institute for health services research (NIVEL) using a survey from 29th of October to 26th of November 2015The Dutch Health Care Consumer Panel consists of more than 10.000 citizens aged 18 years or older in the Netherlands who voluntarily, after invitation by NIVEL, participate in surveys on diverse topics in health care. A sample of 2.250 panel members representative for the general adult population in the Netherlands in terms of age and gender was selected. The study size was chosen to enable collecting information among the whole sample of the general public and among specific subgroups, such as caregivers in this study. These panel members were invited to participate. To reduce the possible selection bias of using only online invitations to participate in the study, participants and received either a questionnaire by email or by post, according to their preferences. A reminder to participate was sent after two weeks for the postal survey and for online participants one and two weeks after initial mailing. In total, 1.097 respondents filled in the survey (response rate 49%).

The questionnaire contained information on normative views of providing care, information on the current provision of informal care, and information on health and general background characteristics of the respondents (see ‘Additional file 1’ for the questionnaire). The questionnaire was reviewed for relevance by the panel committee of the Dutch Health Care Consumer Panel of the Netherlands consisting of representatives of the Dutch Ministry of Health, Welfare and Sport, the Health Care Inspectorate, the Association of Health Care Insurers in the Netherlands, the National Health Care Institute, the Federation of Patients and Consumer Organisations in the Netherlands, the Dutch Healthcare Authority and the Dutch Consumers Association.

### Dependent variable: Views on responsibility for care

The outcome variable assessed views on responsibility for care for persons with a need for care. First information was presented to respondents explaining that ‘people sometimes need assistance or support, such as help with household tasks or nursing care. The government can arrange this care, but the government can also place the responsibility for this care to the general public’. After that the following question was posed: Could you please indicate to whom the responsibility should be placed for (different types of) care for people needing assistance or care? This question was posed for eight prevalent types of care activities (e.g., [1]: personal care (e.g., assisting with bathing or dressing), assistance with taking medication, nursing care (e.g., wound care), household activities (e.g., cleaning, doing the laundry or getting groceries), support with administrative tasks (e.g., submit an application for care or making a doctors’ appointment), support with visits (e.g., accompanying visits to family, doctors or shops), emotional support (e.g., listening), and supervision (e.g., watching the care recipient). Respondents could answer on a 5 point scale with answering categories ‘exclusively the responsibility for the general public’, ‘mainly the responsibility for the general public’, ‘responsibility partly of the general public and partly of the government’, ‘mainly the responsibility for the government’, and ‘exclusively the responsibility for the government’. Answers on these eight care tasks were summed and averaged to represent views on responsibility for care in general (Cronbach’s alpha: 0.85). To investigate differences in views among more care centered activities (e.g., personal care) and activities more towards practical and emotional support (e.g., household activities), we used confirmatory factor analysis with promax rotation to create two sub scores of views (Kaiser–Meyer–Olkin measure of sampling adequacy: 0.85). The sub score of ‘personal or nursing care’ was based on the scores of three care tasks: personal care, assistance with taking medication, and nursing care (alpha 0.81). The sub score of ‘support activities’ was constructed using five tasks: household activities, support with administrative tasks, support with visits, emotional support and supervision (alpha 0.84). All three sum scores on views of responsibility ranged from 1 to 5, with higher scores representing more responsibility for the government.

The questionnaire also included a question on whether respondents would be willing to provide informal care to someone in their social network in the future (answering categories no, yes to someone inside my own household, yes to someone outside my own household). These categories were dichotomized into ‘yes (including ‘yes to someone inside my own household, yes to someone outside my own household’ and ‘no’).

### Independent variables

#### Health

Health of respondents was measured with a question on subjective health with five answering categories (excellent, very good, good, moderate, and bad) which were categorized in ‘(very) good/excellent’ and ‘moderate/bad’. The physical disability level of respondents was measured with a self-reporting disability scale with four classifications: no, mild, moderate or severe physical disability [40]. Moderate disability is defined as having problems with various activities, not only in household tasks but also in mobility. Severe disability is defined as being unable to perform at least one activity independently, that is, needing support. People with severe disability also report problems with self-care activities.

#### Informal care situation

Informal care was defined as care or support to a person with a care need in the social network of the respondent. Informal care could be provided both to persons living outside or inside the household of the respondents. For caregivers sharing a household with the care recipient, it was stated that normal care activities for healthy family members, such as preparing food, are not considered as informal care, and that caregiving should have been provided for at least three months or for eight hours per week or more. Using this information we created a variable indicating whether respondents provided informal care to someone in their social network, classified as informal caregiver at present: no, yes (caregiver for someone in own household and/or caregiver for someone outside household). Among caregivers, subjective burden of caregiving was measured with the CarerQol instrument [41, 42]. Subjective burden was measured with two positive and five negative aspects of caregiving with three response levels: no, some or a lot of. Positive dimensions of caregiving were fulfillment from caregiving and support from others with caregiving. Negative dimensions were relational problems, problems combining daily activities with caregiving, mental health problems, financial problems and physical health problems. A weighted sum score of subjective burden based on the two positive and five negative dimensions of caregiving was calculated. We used a tariff that contained specific weights for each dimension and response level of the CarerQol instrument. This tariff was based on preferences of the general public in the Netherlands for caregiving situations described by the CarerQol instrument. By applying this tariff, a sum score of the CarerQol instrument is calculated that takes differences in the severity of the positive and negative dimensions of caregiving into account. The sum score ranged from 0 to 100, with a higher score indicating less subjective burden of caregiving [43].

#### General background characteristics

The following background characteristics of the respondents were measured: age, gender, educational level in eight different levels classified as ‘low’, ‘middle’ or ‘high’), marital status (married/registered partnership, divorced/widowed or never been married), employment status (fulltime, part-time, no paid work), and monthly household income after taxes categorized into three groups (low <33rd percentile, middle >33rd & <66st percentile, and high >66st percentile of income categories).

### Analysis

Descriptive statistics in percentage or means (standard deviations; SD) were calculated for all variables in this study (Tables 1 and 2). The frequencies of the outcome variables in Table 2 were weighted to adjust for differences in the age and gender of the respondents with that of the general population in the Netherlands.

**Table 1:** Background characteristics, health and informal care characteristics of study sample and the willingness to provide informal care in the future (*n* = 1097)

Background characteristics		
Age (mean. SD)		56.3 (17.2)
Gender (%)	female	52%
	male	48%
Educational level	low	18%
	middle	54%
	high	28%
Marital status	married/reg. Partner	62%
	divorced/widowed	15%
	never been married	23%
Income	low	31%
	middle	36%
	high	33%
Employment status	fulltime	25%
	part-time	23%
	no paid work position	52%
Experienced health		
Subjective health	(very) good/excellent	83%
	moderate/bad	17%
Level of physical disability	No	61%
	Mild	20%
	Moderate/Severe	19%
Informal care situation		
Informal caregiver at present (yes)		28%
Relationship between caregiver and care recipient^b^	Partner	16%
	Parent	31%
	Parent(−in-law)	11%
	Brother/sister	6%
	Child	8%
	Other family	12%
	Friend	9%
	Neighbor	20%
	Acquaintance	19%
Willing to provide informal care in the future		
Willing to provide care	Yes^a^	67%
Types of care wiling to provide^c^:	Personal care	40%
	Assistance with taking medication	61%
	Nursing care	25%
	Household activities	68%
	Support with administrative tasks	72%
	Support with visits	78%
	Emotional support	83%
	Supervision	79%

^a^Includes respondents that chose answering category ‘Yes, to someone inside my own household’ and/or ‘Yes, to someone outside my own household’ (Respondents could choose more than one answering category)

^b^Only respondents who are informal caregivers at present. Percentages sum up to over 100% as some caregivers lend care to more than one care recipient

^c^Only respondents who are willing to provide care in the future. Percentages sum up to over 100% as respondents could choose more than one care task they would be willing to provide in the future

**Table 2:** Descriptives of the views of the responsibility for care tasks

		Responsible for care tasks is (in %):							
		Exclusively the general public	Mainly the general public	Partly the general public and the government	Mainly the government	Exclusively the government	Mean score (sd)	Range	N
Views on responsibility for care in general							2.8 (0.6)	1–5	1009
Views on responsibility for personal or nursing care							3.4 (0.8)	1–5	1018
Personal care		5%	11%	48%	23%	13%			1036
Assistance with taking medication		5%	21%	43%	19%	12%			1033
Nursing care		2%	3%	29%	39%	26%			1033
Views on responsibility for support activities							2.5 (0.7)	1–5	1027
Household activities		5%	22%	55%	12%	6%			1045
Support with administrative tasks		8%	50%	33%	2%	2%			1037
Support with visits		12%	51%	33%	2%	2%			1042
Emotional support		16%	45%	34%	2%	3%			1040
Supervision		12%	39%	42%	4%	4%			1043

*sd* standard deviation

Multivariate association between views on responsibility for care with background characteristics, health and informal care characteristics was performed with ordinary least regression (OLS) reporting standardized coefficients and *p*-values (Table 3). Views of respondents for the three dependent variables care in general (model A), personal or nursing care (model B), and support activities (model C) have been analyzed separately with background characteristics, health and informal care characteristics as independent variables. Among the subgroup of caregivers, OLS regression was performed for views on responsibility for care and subjective burden of caregiving (Table 4). Three models were included for views on care in general (model D), personal or nursing care (model E), and support activities (model F). Background characteristics and health of caregivers were also included in these models. For validation of the results of the OLS models assuming a normal distribution of the dependent variables, the models were analyzed with ordered logistic regression. The models were inspected for multicollinearity among the independent variables using the variance inflation factor. Analyses have been performed in Stata 14.1.

**Table 3:** Multivariate statistics of views on responsibility for care and background characteristics, health and informal care characteristics according to type of care (care in general; model A, personal or nursing care; model B, and support activities; model C)

		Views on responsibility for^a^:					
		MODEL A		MODEL B		MODEL C	
		Care in general (*n* = 930)		Personal or nursing care (*n* = 937)		Support activities (*n* = 947)	
		St. coeff.	*P*-value	St. coeff.	*P*-value	St. coeff.	*P*-value
Background characteristics							
Age		−0.13	0.004	−0.12	0.006	−0.10	0.020
Gender (ref. male)	female	0.12	0.000	0.10	0.004	0.11	0.001
Educational level (ref. high)	low	0.14	0.000	0.11	0.007	0.13	0.001
	middle	0.09	0.021	0.06	0.121	0.09	0.016
Marital status (ref. married/reg. partner)	divorced/widowed	0.01	0.854	0.00	0.912	0.02	0.668
	never been married	0.09	0.017	0.09	0.014	0.07	0.066
Income (ref. high)	low	0.07	0.079	0.07	0.116	0.06	0.172
	middle	0.08	0.042	0.10	0.007	0.04	0.332
Employment status (ref. no paid work)	fulltime	0.04	0.342	0.06	0.186	0.02	0.649
	part-time	0.10	0.013	0.13	0.002	0.05	0.185
Health							
General health (ref. (very) good/excellent)	moderate/bad	0.05	0.137	0.04	0.282	0.05	0.149
Level of physical disability (ref. no)	mild	−0.02	0.528	−0.03	0.344	−0.01	0.884
	moderate/severe	0.07	0.077	0.02	0.526	0.03	0.08
Infomal care characteristics							
Informal caregiver at present (ref. no)	yes	0.03	0.331	0.04	0.202	0.01	0.781
Constant (coef.)		2.73	0.000	3.11	0.000	2.26	0.000
Adjusted R2		0.10		0.08		0.06	

^a^Higher scores indicate more responsibility for the government

**Table 4:** Multivariate statistics of views on responsibility for care *among current caregivers* and background characteristics, health and informal care characteristics, according to type of care (care in general; model D, personal or nursing care; model E, and support activities; model F)

		Views on responsibility for^a^:					
		MODEL D		MODEL E		MODEL F	
		Care in general (*n* = 223)		Personal or nursing care (*n* = 225)		Support activities (*n* = 226)	
		St. coeff.	*P*-value	St. coeff.	*P*-value	St. coeff.	*P*-value
Background characteristics							
Age		−0.09	0.285	−0.01	0.251	−0.05	0.536
Gender (ref. male)	female	0.24	0.000	0.15	0.030	0.26	0.000
Educational level (ref. high)	low	0.28	0.001	0.26	0.002	0.20	0.019
	middle	0.16	0.039	0.12	0.138	0.13	0.094
Marital status (ref. married/reg. partner)	divorced/widowed	0.03	0.677	−0.02	0.756	0.06	0.365
	never been married	0.17	0.010	0.13	0.054	0.15	0.030
Income (ref. high)	low	−0.04	0.648	−0.04	0.674	−0.02	0.810
	middle	0.05	0.542	0.03	0.675	0.06	0.459
Employment status (ref. no paid work)	fulltime	0.13	0.091	0.14	0.092	0.09	0.271
	part-time	0.07	0.341	0.10	0.207	0.02	0.787
Health							
General health (ref. (very) good/excellent)	moderate/bad	−0.02	0.758	−0.03	0.654	0.00	0.984
Level of physical disability (ref. no)	mild	−0.06	0.404	−0.12	0.098	0.01	0.934
	moderate/severe	0.14	0.088	0.09	0.277	0.14	0.084
Infomal care characteristics							
Subjective burden^b^		−0.17	0.029	−0.17	0.030	−0.12	0.139
Constant (coef.)		3.16	0.000	3.86	0.000	2.25	0.000
Adjusted R2		0.17		0.11		0.12	

^a^Higher scores indicate more responsibility for the government

^b^Higher score indicates lower burden of caregiving

## Results

### Study sample

Table 1 presents background characteristics, health and informal care characteristics of the study sample. The mean age of the respondents was 56 years and 52% was female. Most respondents had a middle (53%) or high (28%) educational level. More than 60% of the respondents was married. Of the respondents 52% had no paid work position, 25% had a fulltime and 23% a part-time position.

83% of the respondents perceived their health as (very) good or excellent. Over 60% had no problems with performing physical activities and 19% was not able to perform at least one physical activity by him- or herself.

Of our sample, 28% was an informal caregiver. These caregivers often lent care to a parent(−in-law) (42%), partner (16%), or non-family members like a neighbor (20%) or acquaintance (19%).

### Descriptives of responsibility for different care activities for persons with a need for care

Almost 50% of the respondents stated that the responsibility for personal care activities was a shared responsibility for the general public and the government (Table 2). 36% perceived personal care mainly or exclusively as a responsibility for the government, 31% stated that the government was mainly or exclusively responsible for assistance with taking medication. For nursing care activities, 65% of the respondents stated that the government was mainly or exclusively the responsibility for the government.

In our sample, 55% stated that both the general public and the government are responsible for household activities. About 40% perceived supervision as a shared responsibility for the general public and the government, 51%as a responsibility mainly or exclusively of the general public. Around 60% of the respondents stated that the general public is mainly or exclusively responsible for administrative tasks, support with visits and supervision.

In our sample, 67% stated that they would be willing to provide informal care in the future, when necessary (Table 1). Of those willing to provide informal care, many reported that they would be willing to take up emotional support (83%), supervision (79%), support with visits (78%), support with administrative tasks (72%), and household activities (68%). Concerning personal or nursing activities, 61% of the respondents willing to provide informal care in the future would assistant with taking medication, 40% would provide personal care, and 25% would provide nursing care.

### Multivariate analysis of care for persons with a need for care

*(i) Care in general for persons with a need for care*

Table 3 (model A) shows that younger age, being female, having a lower or middle educational level, never been married, having a middle income, working part-time were associated with perceiving the government as more responsible than the general public for care in general. Both subjective health in general and physical disability were not associated with views on care in general.

*(ii) Personal or nursing care for persons with a need for care*

Younger respondents, females, those with lower educational levels, persons who have never been married, persons with lower income, and those who perform paid work part-time perceived the government more responsible than the general public for personal or nursing care than others. Subjective health (both health in general and physical disability) was not associated with views on personal or nursing care (Model B; Table 3).

*(iii) Support activities for persons with a need for care*

In model C in Table 3, age is negatively associated with perceiving the government mainly responsible for support activities. In addition, females and those with a low or middle educational level perceived the government as more responsible for support activities than others. Views on support activities were not associated with subjective health.

### Views on responsibility for care among a subgroup of current caregivers

Caregivers with a better caregiving situation in terms of subjective burden perceived the government less responsible for care in general and for personal or nursing care activities than burdened caregivers (Model D and E in Table 4). There was not a statistically significant difference between views on responsibility for support activities and the experienced burden of caregiving (Model F; Table 4). Male caregivers, caregivers with a lower educational level, and caregivers who have never been married perceived the government more responsible for all types of care than others (Table 4).

## Discussion

This paper addressed which views the general public and caregivers in the Netherlands hold on the responsibility for the general public versus the government for people who need care. This paper showed that the majority of the general public in the Netherlands would be willing to provide informal care, and especially support activities, in the future, when necessary. Moreover, our results showed that views on who is responsible for care differed per type of care needed. People perceived that informal caregivers like family, friends or neighbors hold more responsibility than the government when support activities were needed. For personal or nursing care the opposite seems to apply. In general, people stated that the government holds more responsibility for personal or nursing care than the own social network of the care recipients. These findings are in line with other research, indicating that many people are willing to help in the future, and that views on the responsibility for elderly care seems to shift from the government to the own social network of elderly persons [29, 34]. However, this does not necessarily apply to addressing all health needs. Although people are willing to help, many also state that they prefer to be incidentally involved in caregiving especially for assisting non-family members with doing groceries or household tasks [1, 25].

Concerning our hypothesis on the role of past experiences with care, we did not find evidence for our hypothesis that persons with more health needs themselves have more preferences for government care. For our hypothesis that informal care characteristics also shaped views on responsibility for care we did find evidence among caregivers. Caregivers with better caregiving situations in terms of experienced burden addressed more responsibility for care to the general public. Here, also the type of care activities was important. Less burdened caregivers viewed the general public more responsible for especially personal and nursing care. Finally, in line with other studies (e.g., [28, 29, 34, 44], this study showed that views on responsibility for care differed among groups of people. Older persons, males, lower educated persons, persons that have never been married and persons working part-time seem to place more responsibility on the government for providing care to persons with a need for care.

When discussing these results, it should be noted that this study is concerned with views and stated preferences. Hence, whether respondents will actually provide care to their family, friends or neighbors in the future could not be concluded from this study. It is merely an indication of the willingness of lending care to persons in their social network in the future. Previous studies have shown that attitudes towards caring are not necessarily strong predictors of actual provision of care, and that this actual behavior of respondents depends among other things on the need for care and preferences for types of care expressed by family, friends or neighbors and supply factors, such as the availability of formal health care services in a country or country-specific eligibility criteria for access to formal health care, the health of respondents themselves in the future, and cultural factors, such as strength of family ties, motivations to care, and norms on family responsibility [3, 19, 23, 28, 31, 45–47]. Furthermore, others have pointed to the trade-off persons often need to make when dividing their time between different activities. For example, employment negatively affects both the willingness of people to provide care and the actual provision of informal care [48]. The willingness to provide informal care in the future depends, as this study underlines, also on the intensity of the demands of the caregiving situation. It is well-known from previous studies that current caregivers often experience caregiving as a straining activity [1–11]. As this study showed, caregivers experiencing strain more often consider the government to be responsible for caregiving. Although we could not directly investigate this, this seems to be an indication that there are also limits in the caregiving tasks that can be handed over to family members and friends. Furthermore, it is important to state that although many respondents felt responsible for caregiving and were willing to provide informal care in the future, it remains uncertain whether these respondents will be willing and able to lend care over longer periods of time.

### Study limitations

The main limitation of this study is the selection effect of using panel data when analyzing views on responsibility for care. First of all, there is a selection in people willing to respond to this survey. Besides, a well-known limitation of panel data is that respondents that are relatively often active on a social and societal level are also expected to participate in research on subjects such as the health care system more often. Furthermore, in this survey the response rate was 49%. Hence, both could have influenced our findings on the willingness to participate in care activities for persons needing care in their social network.

Another limitation of this study concerns the time lag between the time of data collection of the main variables of interest in this study and some background characteristics of respondents, such as their educational level, income, and subjective health. Information on these background characteristics have been collected at the time of becoming a member of the Dutch Health Care Consumer Panel, and have been updated since, whenever possible. However, most of these variables are not expected to change at a very high pace, such as income or educational level. Concerning subjective health which is expected to change more over time, the time lag is 5 or 6 years for 7% of our respondents. For almost 50% of the respondents information on subjective health was collected during the preceding two years and for 16% data was collected in the same year as the survey used in this paper. An additional measure of health, physical disability, was included in the current survey and has also been included in our analyses. Nevertheless, because some characteristics were not gathered at the same time as the outcome measure for this study, some of our results could have been influenced by this time lag.

It also needs noting that only some aspects of the care situation, such as the type of care or the burden of caregiving, were studied. It would be interesting to investigate in further studies whether other aspects of the care situations, such as the intensity, duration of caregiving or the type of relationship with the care recipient, are also relevant in this context. Furthermore, this study investigated views on care in the Netherlands. Besides individual factors, such as gender or educational level, which are associated to views on care (e.g., [20, 28, 44], national factors, such as culture or the formal health care system, also seem to influence these views [27]. For example, support for elderly care provided by the governments is stronger among residents of countries in Northern Europe with an universal long-term care system [20, 27, 34]. Hence, it would be interesting to study care preferences in other countries with different family structures or health care systems than the Netherlands.

## Conclusions

Given the shift in responsibility for care from the government to the general public in many Western European countries, it is essential that a sufficient number of informal caregivers like family, friends or neighbors are willing and able to lend care. To sufficiently fulfill the needs of elderly persons, chronically ill, or disabled persons it is important for policy makers to realize that people in general are willing to assist their family, friends or neighbors when faced with health care needs, but that this willingness depends on the type of care that is needed. Hence, a shift in responsibility for activities more concerned with supporting family, friends or neighbors with household activities or social support is more in line with public views than relying more on family, friends or neighbors to take up care tasks concerned with nursing or personal care. Also, caregivers currently involved in caregiving that feel burdened by this task more often view personal or nursing care as the responsibility for the government than less burdened caregivers. Hence, a critical view on which care tasks could, partly, be handed over to the public domain is needed by policymakers, also in the light of the high burden already imposed on some caregivers at present, and to preserve the quality of the care that is provided to elderly persons, chronically ill or disabled persons.

## Additional file

Additional file 1: Questionnaire_Responsibility for Care. Information on the survey questions used in this study. (DOCX 34 kb)

## Abbreviations

NIVEL: Netherlands institute for health services research
OLS: ordinary least regression
sd: standard deviation
