# Supplementary material for: Who is responsible for providing care? Investigating the role of care tasks and past experiences in a cross-sectional survey in the Netherlands
Source: BMC Health Serv Res. 2017 Jul 11;17:477. doi: 10.1186/s12913-017-2435-5 (PMC5505036; doi:10.1186/s12913-017-2435-5)
Supplement: Additional file 1: — Questionnaire_Responsibility for Care. Information on the survey questions used in this study. (DOCX 34 kb) [file 12913_2017_2435_MOESM1_ESM.docx]

**Questionnaire**

Informal care is care or support that you provide to someone in your own social network.

Do you provide (informal)care to someone outside your own household?

❑ No

❑ Yes, … hours per week

Have you been providing (informal) care to someone in your own household for at least three months or for eight hours per week?

*Normal care activities for healthy family members, such as preparing food, are not considered as informal care. Informal care concerns care in addition to these normal care activities, for example for a chronically ill partner or a child with a disability.*

❑ No

❑ Yes, … hours per week

Which care activities do you provide as a caregiver? *(More than one answer possible)*

| - Personal care (e.g., assisting with bathing or dressing) |
| --- |
| - Assistance with taking medication |
| - Nursing care (e.g., wound care) |
| - Household activities (e.g., cleaning, doing the laundry or getting groceries) |
| - Support with administrative tasks (e.g., submit an application for care or making a doctors’ appointment) |
| - Support with visits (e.g., accompanying visits to family, doctors or shops) |
| - Emotional support (e.g., listening) |
| - Supervision (e.g., watching the care recipient) |
| - Other,………………………. |

To whom do you provide (informal) care in the future? *(More than one answer possible)*

❑ Partner

❑ Brother/sister

❑ Child

❑ Parent

- Parent-in-law
- Other family member

❑ Friend

❑ Neighbour

❑ Another person that I know well

We would like to form an impression of your caregiving situation.

Please tick a box to indicate which description best fits your caregiving situation at the moment.

*Please tick only one box per description: ‘no’, ‘some’ or ‘a lot of’.*

|  | no |  | some | a lot of |  |
| --- | --- | --- | --- | --- | --- |
| I have | ❑ |  | ❑ | ❑ | fulfilment from carrying out my care tasks. |
|  |  |  |  |  |  |
| I have | ❑ |  | ❑ | ❑ | relational problems with the care receiver *(e.g., he/she is very demanding*  *or behaves differently; we have communication problems).* |
|  |  |  |  |  |  |
| I have | ❑ |  | ❑ | ❑ | problems with my own mental health *(e.g., stress, fear,* *gloominess, depression, concern about the future).* |
|  |  |  |  |  |  |
| I have | ❑ |  | ❑ | ❑ | problems combining my care tasks with my own daily activities  *(e.g. household activities, work, study, family, leisure activities).* |
|  |  |  |  |  |  |
| I have | ❑ |  | ❑ | ❑ | financial problems because of my care tasks. |
|  |  |  |  |  |  |
| I have | ❑ |  | ❑ | ❑ | support with carrying out my care tasks, when I need it *(e.g., from family,*  *friends, neighbours, acquaintances)*. |
|  |  |  |  |  |  |
| I have | ❑ |  | ❑ | ❑ | problems with my own physical health *(e.g., more often sick, tiredness, physical stress).* |

How happy do you feel at the moment?

Please place a mark on the scale below that indicates how happy you feel at the moment.

| completely  unhappy | | | | | | | |  | |  | |  | |  | | completely happy | | | | | |
| --- | --- | --- | --- | --- | --- | --- | --- | --- | --- | --- | --- | --- | --- | --- | --- | --- | --- | --- | --- | --- | --- |
|  |  |  |  |  |  |  |  |  |  |  |  |  |  |  |  |  |  |  |  |  |  |
|  |  |  |  |  |  |  |  |  |  |  |  |  |  |  |  |  |  |  |  |  |  |
|  |  |  |  |  |  |  |  |  |  |  |  |  |  |  |  |  |  |  |  |  |  |
|  |  |  |  |  |  |  |  |  |  |  |  |  |  |  |  |  |  |  |  |  |  |
| 0 | | 1 | | 2 | | 3 | | 4 | | 5 | | 6 | | 7 | | 8 | | 9 | | 10 | |

Would you be willing to provide (informal) care to someone in your own household or to someone outside your own household in the future? For caregivers sharing a household with the care recipient, normal care activities for healthy family members, such as preparing food, are not considered as informal care. Informal care concerns care in addition to these normal care activities, for example for a chronically ill partner or a child with a disability. *(More than one answer possible).*

❑ No

❑ Yes, to someone inside my own household

❑ Yes, to someone outside my own household

To whom would you be willing to provide (informal) care in the future? *(More than one answer possible)*

❑ Partner

❑ Brother/sister

❑ Child

❑ Parent

- Parent-in-law
- Other family member

❑ Friend

❑ Neighbour

❑ Another person that I know well

Which care activities would be willing to provide in the future? *(More than one answer possible)*

| - Personal care (e.g., assisting with bathing or dressing) |
| --- |
| - Assistance with taking medication |
| - Nursing care (e.g., wound care) |
| - Household activities (e.g., cleaning, doing the laundry or getting groceries) |
| - Support with administrative tasks (e.g., submit an application for care or making a doctors’ appointment) |
| - Support with visits (e.g., accompanying visits to family, doctors or shops) |
| - Emotional support (e.g., listening) |
| - Supervision (e.g., watching the care recipient) |
| - Other,………………………. |

People sometimes need assistance or support, such as help with household tasks or nursing care. The government can arrange this care, but the government can also place the responsibility for this care to the general public. Could you please indicate to whom the responsibility should be placed for (different types of) care for people needing assistance or care?

|  | Exclusively the responsibility for the general public | Mainly the responsibility for the general public | Responsibility partly of the general public and partly of the government | Mainly the responsibility for the government | Exclusively the responsibility for the government |
| --- | --- | --- | --- | --- | --- |
| Personal care (e.g., assisting with bathing or dressing) | ❑ | ❑ | ❑ | ❑ | ❑ |
| Assistance with taking medication | ❑ | ❑ | ❑ | ❑ | ❑ |
| Nursing care (e.g., wound care) | ❑ | ❑ | ❑ | ❑ | ❑ |
| Household activities (e.g., cleaning, doing the laundry or getting groceries) | ❑ | ❑ | ❑ | ❑ | ❑ |
| Support with administrative tasks (e.g., submit an application for care or making a doctors’ appointment) | ❑ | ❑ | ❑ | ❑ | ❑ |
| Support with visits (e.g., accompanying visits to family, doctors or shops) | ❑ | ❑ | ❑ | ❑ | ❑ |
| Emotional support (e.g., listening) | ❑ | ❑ | ❑ | ❑ | ❑ |
| Supervision (e.g., watching the care recipient) | ❑ | ❑ | ❑ | ❑ | ❑ |
